# Anterior Cruciate Ligament Reconstruction with Quadrupled Semitendinosus Graft or Synthetic Ligament: Knee Stability and Clinical Outcomes at Three Years Follow-Up

**DOI:** 10.1155/2023/4022441

**Published:** 2023-07-20

**Authors:** Lorenzo Moretti, Giuseppe D. Cassano, Alessandro Caricato, Elio Caiaffa, Matteo D'Aprile, Francesco Angiulli, Antonio Spinarelli, Biagio Moretti, Giuseppe Solarino

**Affiliations:** Orthopaedic and Trauma Unit, Department of Basic Medical Sciences, Neuroscience and Sense Organs, School of Medicine, University of Bari Aldo Moro, AOU Consorziale Policlinico, 70124 Bari, Italy

## Abstract

The incidence of anterior cruciate ligament reconstruction (ACLR) surgeries is increasing and graft choice is important for a rapid return to activity, especially in patients older than 30 years. The aim of this study is to compare in term of quality of life and knee stability of patients who undergone ACLR using quadrupled semitendinosus (ST4) graft against patients who undergone ACLR with synthetic ligaments. Thirty-nine patients undergoing ACLR were enrolled in the study and were divided into two groups: ACLR with synthetic ligaments-LARS (group A) and ACLR with quadrupled semitendinosus graft ST4 (group B). They underwent surgery at Policlinico di Bari Orthopedic Unit between January 2017 and January 2020. Group A was composed by nineteen patients (36.16 ± 4.41 mean age-years, 22.47 ± 2.63 mean BMI-kg/m^2^, 39.37 ± 10.05 mean time evaluation after surgery-months) and group B was composed by twenty patients (34.95 ± 3.59 mean age-years, 21.1 ± 2.88 mean BMI-kg/m^2^, 36.75 ± 8.69 mean time evaluation after surgery-months). For each patient, the following data were recorded: age; side of injury, BMI, date of surgery, anterior knee laxity with the arthrometer, and Lysholm knee scoring scale. Mean value of anterior tibial translation (ATT) in group A was 3.09 mm ± 0.65 and in group B was 2.66 mm ± 1.61 (*p*value of 0.1139). Mann–-Whitney *U* test used to compare the Lysholm means values between groups showed a *p*value of 0.9307. LARS has comparable clinical and functional outcomes compared with hamstring autografts at short-term of 3 years follow-up. Level of Evidence: IV.

## 1. Introduction

Anterior cruciate ligament (ACL) injuries occur with an estimated annual incidence of 30–78 per 100000 population, and this number is expected to increase among adolescents as the number of young athletes increases [1, 2]. The appropriate management of partial anterior cruciate ligament (ACL) tears is still debated; for these injuries, ACL augmentation has proved to be an effective and safe procedure and should be preferred to ACL reconstruction in partial ACL tears for the tendency to achieve better functional outcomes [3]. ACL reconstruction (ACLR) surgery is considered to be the gold standard clinical treatment method to allow athletes to return to sports and slow down osteoarthritis degeneration [4, 5].

ACLR is also increasing in middle-aged patients, and it has been seen that ACLR may be performed without concern for inferior clinical and arthrometric results compared with younger patients (<50 years) [6, 7].

Many causes of graft failure have been reported in literature [8–10] and graft choice is a modifiable risk factor for surgeons, especially in patients who want to return to sport as soon as possible and in patients older than 30 years old.

There are different techniques for ACL reconstruction and fixation described in literature [11, 12]. The graft used for ACLR could be an autograft or an allograft from human cadavers and animals or a synthetic graft [13]. Autografts are used more frequently and show better results in terms of surgical results and ability to return to sports, even if a growing number of alternative choices for grafts are becoming available for orthopedic surgeons [14]. Quadrupled semitendinosus (ST4) graft has shown good outcomes and comparable to other autologous graft and a lower morbidity considering that the Gracilis tendon is preserved [15, 16].

The stiffness and strength of the autograft may decrease during the ligamentization process [17–19].

Synthetic ligaments became popular since the late 1970s for providing immediate tensile strength and fast rehabilitation without the risks of disease transmission and immunological rejection. They have been used to overcome the stiffness and strength problems by providing tensile strength and reducing donor site morbidity and to allow a faster return to activity; this is important in elderly patients needing a rapid postoperative recovery [20].

A ten-year longitudinal study by Chen et al. showed satisfactory results and failure rates in patients undergoing primary ACL repair using synthetic ligaments and residual care [21].

However, high failure rates and foreign body synovitis limited their use [22, 23]. However, preliminary results for newer-generation devices, specifically the Ligament Augmentation and Reconstruction System (LARS), show lower reported rates of failure, revision, and sterile effusion/synovitis when compared with older devices [24].

Synthetic grafts have been developed to undertake direct ACLRs and indirect reinforcements of Hamstring Tendons or Bone Patellar Bone (BTB) autografts for ACLR.

Recently, Aujla and colleagues in an observational cohort study compared patients subjected to ACLR with autologous hamstrings augmented with the ligament augmentation and patients subjected to ACLR with hamstrings alone, reported higher Tegner scores and higher return-to-sport rates at preinjury levels one year after surgery in the hybrid group [25].

Comparable with other series showed in the literature, the study of Bugelli and colleagues assesses that the use of LARS in ACLR is an excellent option for treating >40-year-old patients requesting rapid return to daily activities/sports also at the first surgery [26].

Few studies in literature compare clinical and functional scores of autograft and LARS and none of these consider ST4 as a graft or in patients >30 years old. The aim of this study is to compare quality of life and knee stability in patients >30 years old and who undergone ACLR using ST4 graft against patients who undergone ACLR with LARS at 3 years of follow-up.

**Figure 1:**
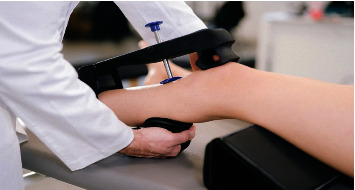
Anterior knee laxity assessment using BLU DAT.F arthrometer.

BLU DAT.F is a knee arthrometer (Figure 1) frequently used to quantify the applied loadings and corresponding tibial anterior displacements; thus, it represents an objective knee laxity evaluation [27].

The aim of this study is to primarily evaluate whether the autograft reduction in stiffness and strength during the ligamentization process in patients older than 30 years old may influence outcomes and therefore assess which between synthetic ligament and autograft should be the first choice in these patients.

## 2. Materials and Methods

### 2.1. Study Design and Sample

This is an observational, retrospective case-control, monocenter study, validated by the Ethics Committee (protocol number: 12/CE/2022—01 May 2022) and performed in accordance with the ethical standards laid down in the 1964 Declaration of Helsinki. All patients involved gave their informed consent prior to their inclusion in the study.

**Figure 2:**
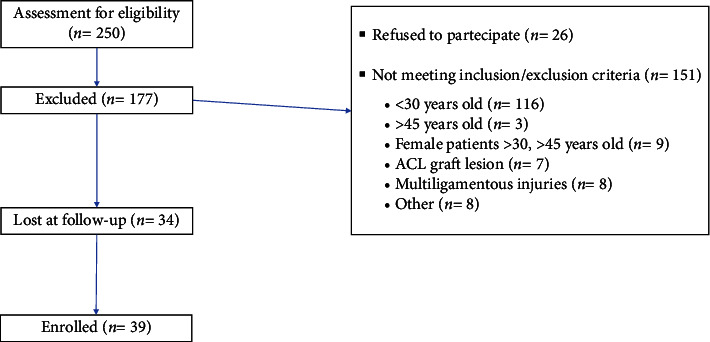
Flow diagram for enrollment and analysis.

Inclusion criteria were male patients undergoing ACLR, age between 30 and 45, negative knee history of major traumatic events after surgery. Exclusion criteria were congenital laxity, combined multiple knee ligament injuries, patients who undergone ACL revision surgery, limited knee range of motion (unable to flex to 20–30°), history of infection, axial deviations, and patients who were not able to understand and complete the procedure due to cognitive dysfunction or language barrier. Two-hundred and fifty patients with ACL lesion were treated by ACLR at the Policlinico di Bari Orthopedic Unit between January 2017 and January 2020. Twenty-six of them refused to participate at the study, one-hundred and fifty-one patients do not respect inclusion and exclusion criteria (one hundred and sixteen patients were <30 years old, 3 were >45 years old, nine female patients were between 30 and 45 years old, seven patients had a new graft lesion following a new trauma, eight patients had multiligamentous injuries, and eight patients were excluded for other reasons such as laxity or axial deviations), and thirty-four were lost at the follow up. Finally, thirty-nine patients were enrolled in the study (Figure 2) and were evaluated at the Policlinico di Bari between August and September 2022.

We choose to enroll male patients excluding female patients' knees in order to obtain a homogeneous sample. As known in literature there are differences in female knee and ACL anatomy, as smaller notch widths and smaller ACL cross-sectional area [28]; female patients undergoing ACLR have been shown to have worse self-reported outcomes, increased risk of contralateral injury, and worse clinical outcomes [29].

Patients enrolled were divided into two groups according to the graft used: ACLR with LARS (group A) and ACLR with ST4 (group B).

For each patient, the following data were recorded: age, side of injury, BMI, date of surgery, anterior knee laxity, and Tegner Lysholm knee scoring scale, as reported in Tables 1 and 2.

### 2.2. Clinical Evaluation

Anterior knee was assessed by a single senior doctor (A.S.) at our outpatient clinic, using the BLU DAT.F arthrometer (FGP BLU DAT.F, Dossobuono, ITA). An 89-N anterior tibial load, at 20° of knee flexion, was applied. At least six measurements for each knee were performed and the median value was registered. The anterior tibial translation (ATT) was expressed in millimeters. ATT value > 5 mm as an indicator for ACLR failure as described in a recent study [30]. After clinical evaluation as secondary endpoint Lysholm questionnaire was administered to evaluate Quality of Life (QoL) and subjective outcomes [31].

The Lysholm Knee Scoring Scale is a patient-reported instrument that consists of subscales for pain, instability, locking, swelling, limp, stair climbing, squatting, and the need for support. Scores range from 0 (worse disability) to 100 (less disability). An assignment is given as “excellent” for 95 to 100 points, “good” for 84 to 94 points, “fair” for 65 to 83 points, or “poor” for less than 65 points.

### 2.3. Surgical Technique

Patients were operated under spinal anesthetic treatment by the same experienced knee surgeon (L.M.), and ACLR all-inside technique was used [32]. Group A patients used LARS ligament as graft. In group B, the semitendinosus tendon used was harvested and prepared as a quadruple graft.

In both groups, a 110° femoral aimer (Femoral ACL Marking Hook for Retro-Construction Drill Guide®—Arthrex©, Naples, FL, U.S.) and a 55° tibial aimer (Tibial ACL Marking Hook) were pointed to the anatomical ACL footprints under direct arthroscopic view. The retrograde femoral half tunnel using FlipCutter® III Drill (Arthrex©, Naples, FL, U.S.) was created, it measured about 2.5 cm. Complete tibial tunnel was created using a 8 mm cannulated drill over a K-wire in group A while tibia half tunnel using FlipCutter® III Drill was created and measured about 2.5 mm length in group B. In group A LARS ligament was duplicated and fixed on the femur side with a suspension system while tibial fixation should be completed with an interference screw at least 1 mm larger in diameter than the tunnel.

In group B, all-inside ACLR was performed with ST4 as described by Cerulli et al. [33] and the graft was fixed with a suspension system both femoral and tibial side. All patients were planned to be discharged on the first day after surgery.

### 2.4. Rehabilitation Protocol

The postoperative protocol for ACLR reconstruction rehabilitation was specific for each group, consistent with the main guidelines in the literature [34, 35]. Rehabilitation started on the first day after surgery and was divided into four phases (immediate in the first month, intermediate, functional, and functional with return to sport).

The immediate phase was the same in the two groups for the first month unless meniscal repairs or cartilage treatments were performed. The articulating knee brace locked in extension was used for the first two weeks and unlocked at 0–45° for another two weeks. Weight bearing was allowed. In the first week passive flexion reached 45°, then active and passive flexion up to 60° was allowed starting from the second week to gradually reach 70° in the ST4 group and 90° in the LARS group at the end of the first month. Isometric quadriceps contractions with the knee extended began after seven days in both groups and were performed at different degrees of flexion starting from week 3. The use of electrostimulation was recommended as was the performance of exercises in a pool after the surgical removal of the surgical sutures. At the end of the first month, the brace was released and gradually abandoned and replaced by a neoprene knee pad.

The intermediate phase which began from the second month gradually involved the complete abandonment of crutches, the execution of semisquats, and the use of the fitness bike and linear walking. The elastic bands and proprioception exercises were helpful.

The third phase, functional phase, for the ST4 group lasted 2 months and involved walking and then linear running, recovery of strength with closed kinetic chain exercises, and then open kinetic chain exercises and proprioceptive exercises. In the LARS group, this phase lasted 1 month. The fourth phase in the ST4 group lasted another two months and included running, jumping, changes of direction, and specific sport exercises; while in the LARS group, it lasted 1 month. Stretching was of fundamental importance in all rehabilitation phases.

Return to sport was foreseen for the 7-8th month in the ST4 group and at the 4th-5th month in the LARS group [17].

### 2.5. Statistical Analysis

Data were collected and analyzed using Microsoft Excel. Categorical variables were presented as numbers or percentages. Continuous variables were presented as mean, median, and standard deviation and Interquartile Range (IQR).

The Shapiro–Wilk test was conducted to verify the normal distribution of the data.

To compare the mean ATT value and mean Lysholm score between groups, the Mann–Whitney *U* test (Wilcoxon rank-sum) was used. A *p*value of <0.05 was considered statistically significant.

Data presented in this study are available on request from the corresponding author.

## 3. Results

Thirty-nine subjects were enrolled in this study and divided into two groups: group A was composed by nineteen patients (36.16 ± 4.41 mean age-years, 22.47 ± 2.63 mean BMI-kg/m^2^, 39.37 ± 10.05 mean time evaluation after surgery-months) and group B was composed by twenty patients (34.95 ± 3.59 mean age-years, 21.1 ± 2.88 mean BMI-kg/m^2^, 36.75 ± 8.69 mean time evaluation after surgery-months).

We compared the study and control group at recruitment. The main demographic characteristics are described in Table 1.

None of the patients experienced any skin complications due to the procedure. None of the patients need for revision surgery due to infection and mechanical failure. Two patients in group B had persistent fever for 3 days after surgery and were discharged on day 4 after surgery.

The ATT expressed in millimiters and Lysholm score were calculated and shown in Table 2. Shapiro–Wilk test showed a non-normal distribution for ATT value and Lysholm score value and so Mann–Whitney *U* test was used to compare ATT and Lysholm score between groups.

In group A, none had ATT values > 5 mm while in group B one patient had translation values > 5 mm and other two patients had a mean value near 5 mm.

**Figure 3:**
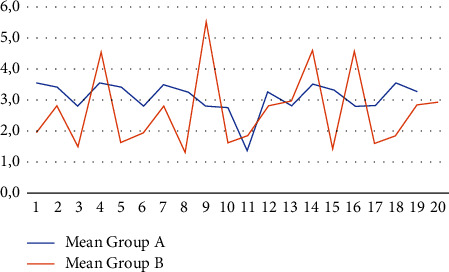
Mean value of ATT expressed in mm in group A and B.

Mean ATT values obtained from the six measurements for each patient are shown in Figure 3.

The Lysholm score in group A was excellent in 16 patients, good in 2 patients, and fair in 1 patient; mean values are shown in Table 2.

## 4. Discussion

The most important findings of this study are that the mean and median ATT values for group B which are results lower than group A but with a higher standard deviation (1.61 vs. 0.65). This shows a greater-scores-variability. Furthermore, no patient of group A (ACLR with LARS) has shown an ATT value that exceeds 5 mm.

Mann–Whitney *U* test used to compare mean ATT value and Lysholm score has shown not statistically significant values therefore the synthetic graft must be taken in consideration as a graft for over 30 -years-old patients.

Results shown in the study are similar to those described by other authors who compared ACLR with Hamstrings Tendons (HT) versus ACLR with LARS.

In 2010, Zhong-tang Liu and colleagues showed better functional outcomes and high knee stability in LARS group compared to Hamstring group at 49 months follow up. LARS group had significantly less anterior displacement than the hamstring group (*p*=0.013). Although other results of ACLR, measured by IKDC evaluation, Lysholm scores and Tegner activity level, showed using a LARS graft were superior to using hamstring, though there were no significant differences [36].

In a retrospective study of Hamido et al., 27 patients treated for chronic ACL lesion with undersized HT graft with LARS-augmentation were compared with 45 patients treated with four-strand hamstring tendon graft (4SHG). In this study, LARS-augmented group had significantly less anterior displacement than the 4SHG group at 5 years follow-up [37].

In both studies, the SD is slightly higher in the group of patients using hamstrings as grafts; in our study, we evaluated a higher SD between groups (1.61 group B vs. 0.65 group A). This difference between groups can be explained by considering LARS's characteristics which has less reduction of stiffness and strength over time.

In 2018, Bianchi and colleagues evaluated clinical, functional, and radiographic outcomes in 50 patients who underwent ACLR (25 4SHG and 25 LARS). The study suggests that the patients in the LARS group displayed a higher-knee stability than 4SHG group [38].

In a recent study with 185 patients was evaluated the clinical efficacy of ACLR with 4SHG, allograft LARS ligament. The authors found no statistically significant differences among the 4SHG, allograft, and LARS ligament in terms of the clinical outcomes after ACLR at 5-years follow-up [39].

Actually, there is still no consensus regarding the optimal graft tissue choice in ACLR and especially in over 30 -years-old active patients.

Latest review shows that QT and HT autografts have comparatively good results in ACLR without significant differences regarding function, pain, and rupture after surgical intervention [40].

Even studies that evaluated the use of BTB as a graft versus LARS did not show statistical significance in the outcomes differences [41], while others indicates that for adults, BPTB grafts perform more favorably than synthetic grafts in ACLR in terms of knee stability, function, and complication [42].

On the other hand, the higher SD value (1.61) in group A may be explained in subject anatomical variability and ST harvesting technique that results in different size and quality of the autograft [43]. Premature amputation of the tendon and a smaller graft is one of the common complications in ACLR with an hamstring autograft, and it may depend on stripper inclination during the harvesting surgery step and may resulting in initial graft tension and outcome [44, 45].

Another factor to consider is the rehabilitation program used in the two groups. As shown in Tables 1 and 2, the use of LARS in the ACLR allows for faster rehabilitation and a shorter return-to-sport time than the use of autograft. Indeed, according to Chen et al. in a prospective cohort study in patients undergoing ACLR with HT autografts (*n* = 73) versus LARS (*n* = 38) with 10 years follow-up; the lack of donor site morbidity in the LARS group could explain early functional outcomes [46].

In our opinion, beyond the graft used, the surgical technique and the rehabilitation program play an important role to reach satisfactory clinical and functional outcomes and are important to know the graft specifics and biological transformations after ACLR for the correct timing to return to activity after injury.

The strengths of this study are the sole surgeon for the patients of both groups under examination, the stringent evaluation of the inclusion and exclusion criteria, and the absence of substantial bias between groups. Our study has some limitations such as the simple number of patient examined and the short follow-up with a high standard deviation; therefore, further studies with a larger number of patients and longer follow-up are therefore necessary in order to assess possible long-term complications and to delineate the reasons for the uneven distribution of data regarding ST4 group (e.g., graft diameter, and technique).

## 5. Conclusions

According to our results, there are no statistically differences in ATT values and clinical outcome assessed with Lysholm score between groups. The ATT evaluation shows a lower mean value in patients who undergone ACLR with ST4 but with a high standard deviation; SD is lower in LARS group and no patient exceeded the cut-off values of 5 mm of translation. In conclusion, after a minimum 3 years after ACLR, comparable and successful clinical and functional outcomes can be expected using ST4 or LARS synthetic ligament as graft in over 30 -years-old male men.

## Figures and Tables

**Table 1:** Baseline evaluation of study participants.

Preoperative features	Group A	Group B	*p*-value
Age (year)	36.16 ± 4.41	34.95 ± 3.59	0.45
BMI (kg/m^2^)	22.47 ± 2.63	21.1 ± 2.88	0.11
Side (left)	9 (47.3%)	11 (55%)	0.75
Time from surgery (months)	39.37 ± 10.05	36.75 ± 8.69	0.45

^*∗*^ *U* Mann–Whitney and Fischer's test; data are presented as mean ± standard deviation or number and percentage; BMI: body mass index. No statistical differences emerged between groups.

**Table 2:** Differences in anterior knee laxity and Lysholm score between groups at the follow-up.

		Group A	Group B	*p* value
ATT	Mean	3.09	2.66	0.1139
	Median	3.10	2.60	
	SD	0.65	1.61	
	IQR	0.6	1.92	

Lysholm score	Mean	93.5	91.9	0.9307
	Median	95	95	
	SD	5.73	8.10	
	IQR	5.5	15	

^*∗*^(ATT = anterior tibial translation, SD = standard deviation, IQR = interquartile 25th–75th percentiles).

## Data Availability

The research data used to support the findings of this study are available from the corresponding author upon request.
